# An online compendium of treatable genetic disorders

**DOI:** 10.1002/ajmg.c.31874

**Published:** 2020-12-22

**Authors:** David Bick, Sarah L. Bick, David P. Dimmock, Tom A. Fowler, Mark J. Caulfield, Richard H. Scott

**Affiliations:** ^1^ HudsonAlpha Institute for Biotechnology Huntsville Alabama USA; ^2^ Nemours/Alfred I. duPont Hospital for Children Wilmington Delaware USA; ^3^ Rady Children's Institute for Genomic Medicine San Diego California USA; ^4^ Genomics England Ltd. London UK; ^5^ William Harvey Research Institute Queen Mary University of London London UK; ^6^ Department of Clinical Genetics Great Ormond Street Hospital for Children National Health Service (NHS) Foundation Trust London UK

**Keywords:** genetic diseases, internet, mobile application, treatment

## Abstract

More than 4,000 genes have been associated with recognizable Mendelian/monogenic diseases. When faced with a new diagnosis of a rare genetic disorder, health care providers increasingly turn to internet resources for information to understand the disease and direct care. Unfortunately, it can be challenging to find information concerning treatment for rare diseases as key details are scattered across a number of authoritative websites and numerous journal articles. The website and associated mobile device application described in this article begin to address this challenge by providing a convenient, readily available starting point to find treatment information. The site, Rx-genes.com (https://www.rx-genes.com/), is focused on those conditions where the treatment is directed against the mechanism of the disease and thereby alters the natural history of the disease. The website currently contains 633 disease entries that include references to disease information and treatment guidance, a brief summary of treatments, the inheritance pattern, a disease frequency (if known), nonmolecular confirmatory testing (if available), and a link to experimental treatments. Existing entries are continuously updated, and new entries are added as novel treatments appear in the literature.

## 1 INTRODUCTION

The etiologies for many rare diseases have been discovered. Online Inheritance in Man (OMIM) (https://omim.org/statistics/geneMap, October 31, 2020) lists 4,339 genes with phenotype‐causing variants. Orphanet (http://www.orphadata.org/cgi-bin/index.php, 10/09/2020) includes more than 7,800 disease‐gene relationships. Each year about 250 rare genetic disease discoveries are added to the lists. These discoveries include pathogenic variants in a gene that had not previously been associated with disease and pathogenic variants in a gene previously associated with a different disease (Boycott et al., 2017).

While the diagnosis of genetic disorders has improved, management has not kept pace. For most genetic disorders, treatment is symptomatic and not directed against the mechanism of disease. For example, many genetic causes of deafness are effectively managed with cochlear implants, a therapy that is not specific to the underlying disorder (Usami, Nishio, Moteki, Miyagawa, & Yoshimura, 2020). Brugada syndrome is managed with an implantable defibrillator, a therapy that is not directed against the underlying cardiac ion channel channelopathies (Brugada et al., 2016). Yet, for other disorders, such as phenylketonuria, treatment of individuals is directed at the underlying pathology and has a dramatic effect on the outcome (Jameson & Remmington, 2020).

When faced with a new diagnosis of a rare genetic disorder, physicians traditionally turned to textbooks and the medical literature for information to direct management. In recent years, however, the value of internet resources in patient care has been documented (Maggio, Aakre, Del Fiol, Shellum, & Cook, 2019). A number of online resources that detail the overall phenotype and management of genetic disorders are now available on a desktop computer or mobile device (OMIM, https://omim.org/; Orphanet, https://www.orpha.net/consor/cgi-bin/index.php; GeneReviews, https://www.ncbi.nlm.nih.gov/books/NBK1116/; Clinical Genomic Database, https://research.nhgri.nih.gov/CGD/search/ (Solomon, Nguyen, Bear, & Wolfsberg, 2013); Genetic and Rare Disease Information Center, https://rarediseases.info.nih.gov/; UpToDate, https://www.uptodate.com/contents/search; ClinGen Actionability Knowledge Repository, https://actionability.clinicalgenome.org/ac/). When considering these disorders, providers pay particular attention to those with a specific treatment based on the underlying molecular pathology. In many instances, early identification, ideally before symptoms appear, leads to a superior outcome. The website described in this article is focused on those conditions where the treatment is directed against the mechanism of the disease and thereby alters the natural history of the disease in at least some of the patients.

## 2 METHODS

All disorders selected for the website and associated mobile device application are Mendelian disorders associated with treatments that affect the underlying pathogenesis and progression of disease. The sources used to identify the genetic disorders include two publications (Lee et al., 2019; Milko et al., 2019), two websites (GeneReviews, https://www.ncbi.nlm.nih.gov/books/NBK1116/; Clinical Genomic Database, https://research.nhgri.nih.gov/CGD/ (Solomon et al., 2013)) and the table of contents from the last 2 years of 45 journals (Appendix S1) reviewed by one curator (DB) for titles that identified treatments of a genetic disorder.

Genetic disorders with a Food and Drug Administration (FDA) approved therapy were included in the website. Additionally, publications were reviewed by a single curator (DB) to evaluate therapies for other genetic disorders. If the article reported an improvement in more than one case the therapy was included in the approaches that a physician should consider.

Evidence of efficacy fell into two categories, those with published therapeutic guidelines, and those without a guideline. The latter were designated “expert opinion,” Guidelines developed by a national society were preferred over those developed by disease specific foundations or expert panels. More recent guidelines were preferred over older guidelines. These were identified by a review of the literature and through web search engines.

**FIGURE 1:**
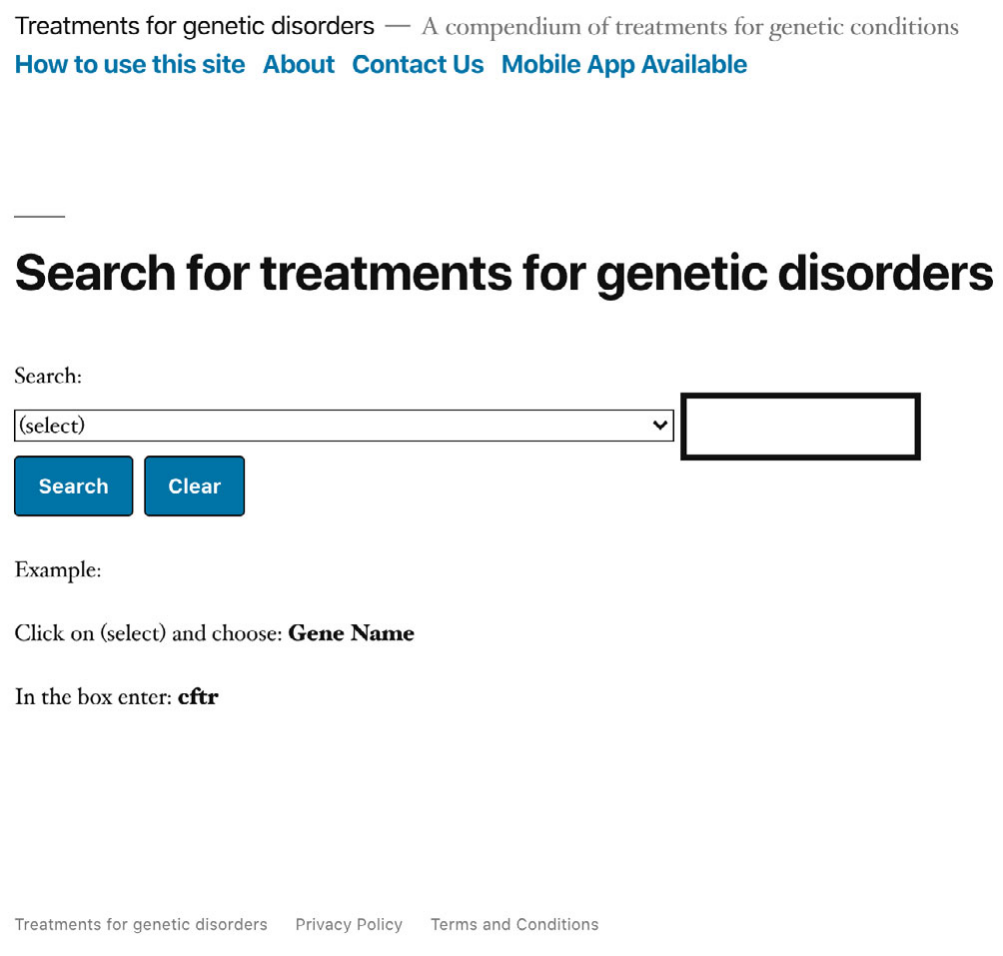
Homepage of Rx‐genes.com (https://www.rx‐genes.com/) website showing the search window that allows the user to perform a search in any field in the database

**FIGURE 2:**
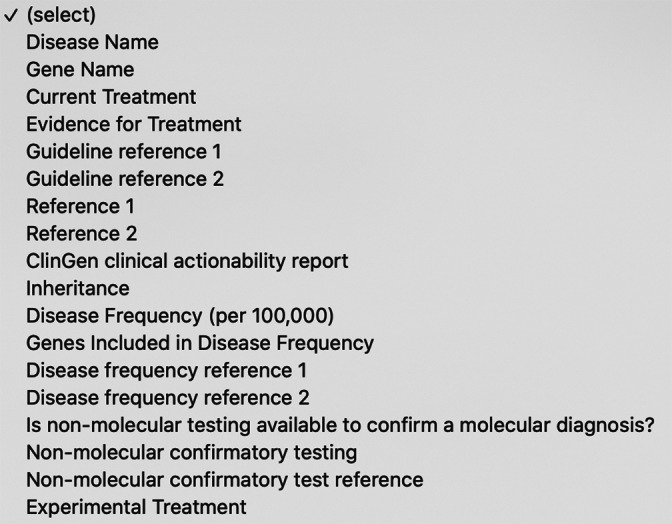
List of each of the fields in the database that can be queried at the Rx‐genes.com (https://www.rx‐genes.com/) website

**FIGURE 3:**
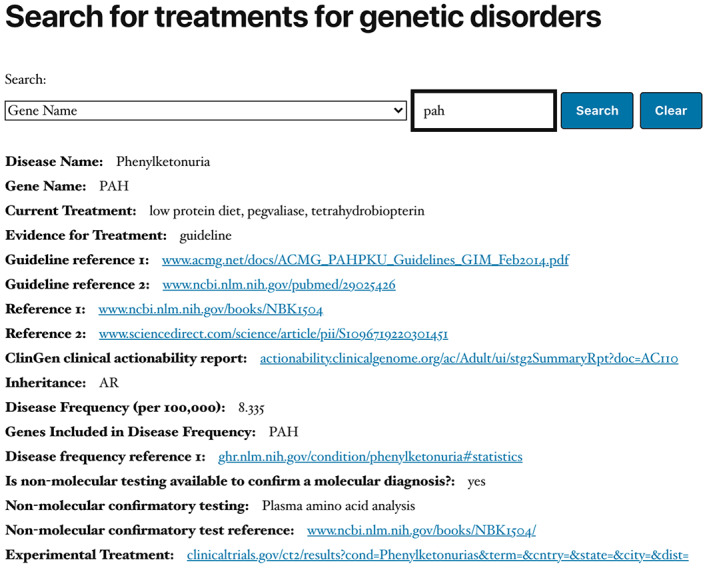
Shows the result of a search in Rx‐genes.com for the gene, PAH. The result displays information and references about the gene and the associated disease

The website and mobile device application provide a simple interface (Figure 1), allowing the user to submit a query based on any field in the database (Figure 2). Each entry is focused on a particular disease and contains a number of relevant data fields described in Table 1. Figure 3 is an example of an entry in the database. Information for every data field is not available for every disease. Some genes are associated with more than one disease.

**TABLE 1:** Description of the data fields for each database entry underlying the Rx‐genes.com (https://www.rx‐genes.com/) website and mobile device application

Data field	Data field description
Disease name	Uses the disease name designation in OMIM where possible.
Gene name	Uses the symbol assigned by the HUGO gene nomenclature Committee at the European Bioinformatics Institute (https://www.genenames.org/ ).
Current treatment	Lists treatment approaches that a physician should consider.
Evidence for treatment	Indicates whether treatment is supported by a published guideline or relies on expert opinion.
Guideline reference 1 and guideline reference 2	Provides references when a disorder has published treatment guidelines.
Reference 1 and reference 2	Provides general information and treatment guidance for the genetic disorder.
ClinGen clinical actionability report	Provides information and treatment guidance for the genetic disorder developed by ClinGen and found in the actionability knowledge repository (https://actionability.clinicalgenome.org/ac/) .
Inheritance	Uses AR for autosomal recessive, AD for autosomal dominant and XL for X‐linked.
Disease frequency	Provides an estimate of the disease prevalence. Where possible a birth prevalence is provided. When there is a range of prevalence figures an average of the highest and lowest values is used.
Genes included in disease frequency	Contains the list of genes included in the disease frequency estimate.
Disease frequency reference 1 and disease frequency reference 2	Provides sources of the prevalence figures.
Is nonmolecular testing available to confirm a molecular diagnosis?	This is answered in the affirmative when the test involves a readily obtained sample such as blood, urine, cerebrospinal fluid, and bone marrow aspirate or involves a noninvasive procedure such as an MRI or ophthalmologic examination. Additionally, the test must be generally available. These tests are particularly important when the patient's phenotype combined with a molecular test that finds a variant of uncertain significance, do not provide enough information for a physician to make a treatment recommendation such as a hematopoietic stem cell transplant. Confirmatory tests performed in research laboratories and assays that require invasive procedures such as skin biopsy, liver biopsy, kidney biopsy, and brain biopsy are not considered as “available” tests as they require special procedures and handling and testing laboratories are not generally available.
Nonmolecular confirmatory testings	Lists the test or tests that can be used to confirm a molecular test.
Nonmolecular confirmatory test reference	Provides a reference for the confirmatory test(s).
Experimental treatment	Provides a link to clinical trials at clinicaltrials.gov (https://clinicaltrials.gov/) for experimental treatments.

## 3 RESULTS

The database underlying the Rx‐genes.com (https://www.rx-genes.com/) website and associated mobile device application currently contains 633 disease entities resulting from variants in 619 genes. Each of 14 genes result in 2 different diseases; 14% (619/4,339) of the 4,339 genes associated with phenotype‐causing variants listed in Online Inheritance in Man (OMIM) (https://omim.org/statistics/geneMap, October 31, 2020) are contained in the database. For 19 of the genes in the database, an adult onset disease is recognized (*UROD*, *SLC30A2*, *BRCA1*, *BRCA2*, *LDLR*, *PCSK9*, *APOB*, *LDLRAP1*, *APOE*, *MLH1*, *MSH2*, *MSH6*, *PMS2*, *EPCAM*, *MUTYH*, *BMPR1A*, *SMAD4*, *CP*, *HFE*).

The diseases included in the database require a wide range of treatments due to the myriad physiologic pathways involved. For genes that share a physiologic pathway there is often a common disease process that is amenable to the same treatment or set of treatments.

### 3.1 Dietary management

Dietary management plays an important role in 95 diseases. For example, limiting dietary galactose is effective for diseases arising from four genes that affect galactose metabolism (*GALT*, *GALE*, *GALK1*, and *GALM*).

### 3.2 Hematopoietic stem cell transplantation

Based on the sources reviewed, hematopoietic stem cell transplantation (HSCT) has been reported to have been used effectively in 157 diseases. Most are disorders arising from dysfunction or failure of one or more stem cell lineages causing disorders such as severe combined immunodeficiency, thalassemia, and congenital amegakaryocytic thrombocytopenia. HSCT is also used to treat genetic abnormalities in *CLCN7*, *TCIRG1*, *SNX10*, and *TNFRSF11A* leading to osteopetrosis. Additionally, HSCT has been used in a number of storage disorders (Krabbe disease, mucopolysaccharidosis (MPS) I, MPS II, MPS VII) as a way to supply the missing enzyme.

### 3.3 Enzyme replacement therapy

Enzyme replacement therapy (ERT) is available for 28 disorders. This includes specific enzyme replacement for disorders associated with 17 genes (*PAH*, *GAA*, *ALPL*, *SERPINA1*, *ADA*, *GLA*, *LIPA*, *TPP1*, *GBA*, *IDUA*, *IDS*, *SMPD1*, *MAN2B1*, *GALNS*, *ARSB*, *NAGLU*, *GUSB*). For diseases associated with 11 genes (*CFTR*, *SBDS*, *DNAJC21*, *EFL1*, *SRP54*, *ABCC8*, *KCNJ11*, *PTF1A*, *GATA6*, *GATA4*, and *PDX1*) pancreatic enzyme replacement may be part of the therapy for the associated disease. Although not strictly a replacement, the enzyme, deoxyribonuclease is used in cystic fibrosis.

### 3.4 Solid organ transplantation

Solid organ transplantation is used to manage 18 disorders including liver transplantation for 12 disorders, kidney transplantation for 4 disorders, heart transplantation for 1 disorder, and thymus transplantation for 1 other disorder.

### 3.5 Supplements

Supplements are important in the treatment of 175 disorders (Appendix S2). These include minerals (ZnSO4 NaCl, KCl, MgCl, CaCl, NaHCO3, Na3PO4, CuCl2, MnSO4), vitamins (vitamin K, vitamin D, vitamin A, vitamin E, pantothenic acid, pyridoxine, riboflavin, cobalamin, folate), sugars (corn starch, galactose, fucose, mannose), and other natural products (creatine, phosphatidylcholine, ornithine, chenodeoxycholic acid, cholic acid, uridine, carnitine, betaine, 5‐hydroxytryptophan, pyruvate, coenzyme Q10, biopterin, taurine, glycine, D,L‐3‐hydroxybutyrate, citrulline, lysine, serine).

### 3.6 Immunoglobulin therapy

Immunoglobulin therapy is used in the management of 42 immunodeficiencies in which agammaglobulinemia, hypogammaglobulinemia, or dysgammaglobulinemia are aspects of the diseases.

### 3.7 Medications, vaccinations, and blood products

Medications, vaccinations, and blood products play a role in the management of 328 disorders. This includes 154 medications, 3 vaccines, and 9 different blood products (Appendix S2). While most medications are specific, some fall into a general category such as antibiotics.

### 3.8 Gene therapy

Gene therapy is approved for four disorders: beta‐thalassemia, spinal muscular atrophy, adenosine deaminase deficiency, and RPE65‐related Leber congenital amaurosis.

### 3.9 Procedures

Medical procedures manage certain aspects of 36 disorders. These procedures include phlebotomy, colonoscopy with polyp removal, endoscopy with polyp removal, apheresis, prophylactic mastectomy, prophylactic oophorectomy, transsphenoidal surgery, thyroidectomy, colectomy, bilateral adrenalectomy, and pancreatic resection. For example, prophylactic mastectomy and prophylactic oophorectomy have been proven to be effective in preventing most breast and ovarian cancer in BRCA1‐ and BRCA2‐associated hereditary breast and ovarian cancer syndrome (Petrucelli, Daly, & Pal, 2016). For multiple endocrine neoplasia type 2 (Eng, 2019) thyroidectomy can prevent medullary thyroid carcinoma.

## 4 DISCUSSION

As more genes are associated with recognizable disorders, an increasing number of healthcare providers, both geneticists and non‐geneticists, are employing genomic testing in patient care. Recognizing this trend, efforts are underway to make the results of these diagnostic tests understandable to non‐specialists (Recchia, Chiappi, Chandratillake, Raymond, & Freeman, 2020). When faced with a genetic question in practice, physicians turn to the internet to acquire “just‐in‐time” information (Evans, Tranter, Rafi, Hayward, & Qureshi, 2020). In response, excellent online tools have been developed to aid in the diagnosis of rare disorders and evaluate the variants found in molecular laboratory reports (Reches, Weiss, Bazak, Feldman, & Maya, 2019). Once a genetic diagnosis is established the patient's healthcare team needs to establish a management plan and, where possible, initiate treatment. Unfortunately, this information is scattered across many internet sites.

### 4.1 Uses of Rx‐genes.com

Rx‐genes.com (https://www.rx-genes.com/) is an initial effort targeted to clinicians in need of “just in time” information when they encounter a newly diagnosed patient in their office or in the hospital. The website and associated mobile device application are focused on information that providers generally seek when faced with a newly diagnosed genetic disorder: (a) authoritative information about the disease, (b) current treatment, (c) disease treatment guidelines (if they exist), (d) inheritance pattern, (e) disease frequency, (f) nonmolecular confirmatory testing (if it exists), and (g) experimental treatment. The website and underlying data can be used in other settings, including genomic laboratory testing, the electronic medical record, and the design of new healthcare or research programs related to newborn screening. It must be emphasized that a treatment information aggregation site is no substitute for clinical judgment, a review of the primary literature and consultation with disease specific experts.

Elective genomic testing (Lu et al., 2019) as an adjunct or replacement for newborn screening is being studied (Adhikari et al., 2020; Ceyhan‐Birsoy et al., 2019; Roman et al., 2020; Trier et al., 2020). The genetic diseases found at this website represent a useful starting point for programs planning to use genomic testing for newborn screening. The site provides a list of genetic disorders where nonmolecular testing is available to confirm molecular test results. This list could be used as an initial gene list for consideration in a newborn screening program because nonmolecular testing may be required before genomic testing can be accepted as a stand‐alone test in certain contexts. For each nonmolecular test, it will be necessary to estimate the positive predictive value of the test in the setting of an asymptomatic newborn with a positive molecular test. The site also provides a disease frequency estimate for 380 of the 633 genes in the compendium including 16 of 19 genes associated with adult onset disorders. Two genes, *BRCA2* and *LDLR*, are each associated with an adult dominant disorder and a recessive childhood onset disorder. These estimates can help programs choose the diseases to target. The sum of the frequencies for the 380 genes is ~1 in 50 (1,989 per 100,000) births. If the 16 adult disorders are removed, the sum of the frequencies of the remaining 364 genes is ~1 in 190 (527 per 100,000) births. One in 190 is obviously an underestimate for the 614 genes that are not associated with an adult disorder given the absence of frequency data for 250 of the genes. To put this in context, there were 625,651 births in England in 2018. (Office for National Statistics, https://www.ons.gov.uk/peoplepopulationandcommunity/populationandmigration/populationestimates/datasets/vitalstatisticspopulationandhealthreferencetables#:~:text=In%202018%2C%20there%20were%20731%2C213,the%20lowest%20rate%20on%20record) Using the figure of 1 in 190 births, this data suggests that 9 children were born each day with a treatable disorder.

Genomic testing reports performed on cancer samples routinely contain variant and gene specific links to standard and experimental treatment options. Laboratories often incorporate information from web‐based resources such as IBM Watson for Genomics, NAVIFY Mutation Profiler, OncoKB, TQuest, and N‐of‐One (Gershkovich et al., 2018; Katsoulakis, Duff, Hintze, Spector, & Kelley, 2020; Yaung, Krishna, Xi, C, & Palma, 2020). Laboratories reporting genomic testing results for rare diseases could incorporate a link to Rx‐genes.com (https://www.rx-genes.com/), where appropriate.

For providers in the hospital setting, the need for “just in time” information delivered through decision support tools in the electronic medical records have been shown to improve patient care (Kwan et al., 2020). Links to the website through an application programming interface could be integrated into genetics decision support in the electronic medical record (https://www.sciencedirect.com/science/article/pii/B9780128006818000141).

### 4.2 Limitations

The website has a number of limitations: (a) Treatments published outside of the 45 English language journals, two articles and two websites reviewed will be missed. (b) Treatments found effective in small cohorts may not prove to be useful in a larger study of affected individuals, were not performed according to “good clinical practice” (The International Council for Harmonization, https://www.ich.org/page/efficacy-guidelines) in most instances and generally used heterogenous control groups, typically historical in nature. (c) Existing treatment guidelines may have been missed by the searches undertaken or may be unavailable (e.g., behind a firewall). For some disorders such as phenylketonuria there are many guidelines beyond those listed on the website. (d) Some treatments have conflicting evidence with respect to outcome. The use of hematopoietic stem cell transplantation in Krabbe disease is an example (Dimmock, 2016). (e) At present the site only includes disorders with a treatment that is associated with the underlying pathogenesis. This is clearly a limited subset of “actionable” disorders that include a large number with anticipatory management guidelines and treatments unrelated to pathogenesis. (Milko et al., 2019; Cehyan‐Birsoy et al., 2019; Reble et al., 2020; Adhikari et al., 2020; Richer & Laberge, 2019; Clinical Actionability Curations, https://www.clinicalgenome.org/curation‐activities/clinical‐actionability/browse‐curations/, https://www.hrsa.gov/advisory-committees/heritable-disorders/rusp/index.html). As the published literature demonstrates, however, there is no general agreement on what constitutes an “actionable” disorder. This problem of assigning actionability to variants and genes also occurs in molecular oncology (Katsoulakis et al., 2020; Mateo et al., 2018; Parikh, Love‐Gregory, Duncavage, & Heusel, 2020). (f) The diseases chosen for inclusion in the website are focused on disorders with a childhood onset. (g) Disease frequency estimates are based on literature for the general population. The site does not provide data for particular ethnic groups and genetic isolates with significantly higher frequencies. (h) Nonmolecular confirmatory testing was narrowly defined (Table 1) including only blood, urine, cerebrospinal fluid, or bone marrow aspirate as the sources of material for the test and noninvasive tests such as magnetic resonance imaging. It did not include skin punch biopsy, a commonly used sample type for numerous genetic tests. While skin biopsy is minimally invasive, its use is a particular problem in newborn screening where parents may be reluctant to agree to the procedure in an apparently healthy infant.

### 4.3 Future considerations

Ongoing development of the website will focus first on disorders that have not been assessed for an associated nonmolecular confirmatory testing and the development of an application programming interface. Early user feedback requested a link to all disease‐gene associations, including those that are not treatable as defined by the website. Ongoing updates of the current catalogue of disorders and identification of new diseases to add to the site represent a special problem. It may be possible to employ a machine learning approach to find new entries and to ensure the site is kept up to date for the existing entries.

### 4.4 Summary

Rx‐genes.com (https://www.rx-genes.com/) represents an initial effort to provide a readily accessible website and mobile device application for clinicians seeking relevant “just‐in‐time” information from the medical literature when faced with an unfamiliar genetic diagnosis. At present, the information is entirely focused on rare disorders where treatment is associated with the underlying pathogenesis of the disease. The site will update existing entries, add treatable disorders, and expand to include actionable disorders more broadly.

## CONFLICT OF INTEREST

D. B. and S. L. B. declare no competing financial interests. D. D. received funding from Biomarin (consultant for Pegvaliase trials), Audentes Therapeutics (Scientific Advisory Board), and Ichorion Therapeutics (consultant for mitochondrial disease drugs). M. J. C., T. A. F., and R. S. are seconded to, and receive salary from, Genomics England Ltd, a wholly owned Department of Health and Social Care Company in the UK.

## AUTHOR CONTRIBUTIONS

David Bick developed the website, contributed to the data underlying the website, drafted the initial article, and revised the article. Sarah L. Bick contributed to the data underlying the website and reviewed the article. David P. Dimmock contributed to the data underlying the website and reviewed and revised the article. Tom A. Fowler contributed to the data underlying the website and reviewed the article. Mark J. Caulfield contributed to the concepts central to the website and reviewed and revised the article. Richard Scott contributed to the concepts central to the website, contributed to the data underlying the website and reviewed the article.

## Supporting information


**Appendix** **1**. Articles found in these journals were reviewed to identify content for the Rx-genes.com (https://www.rx-genes.com/) website.

**Appendix** **2**. List of the 154 medications, 3 vaccines, and 9 different blood products included in the Rx-genes.com (https://www.rx-genes.com/) website.

## Data Availability

The data that support the findings of this study are available from the corresponding author upon reasonable request.
